# Two-years clinical course of pain intensity and symptom satisfaction for latent classes in older adults with back pain

**DOI:** 10.1186/s12891-025-09442-w

**Published:** 2025-12-23

**Authors:** Ann-Christin Sannes, Lise Kretz, Are Hugo Pripp, Ørjan Nesse Vigdal, Rikke Munk Killingmo, Silje Stensrud, Kjersti Storheim, Margreth Grotle, Iben Axén

**Affiliations:** 1 Department of Rehabilitation Science and Health Technology, Oslo Metropolitan University, Pilestredet Park, Oslo, 0890 Norway; 2 ELIB-Et Liv I Bevegelse research foundation, Oslo, Norway; 3 Department for Research and Development in Mental Health, Akershus University Hospital, Lørenskog, Norway; 4 Oslo Centre of Biostatistics and Epidemiology, Research Support Services, Oslo University Hospital, Oslo, Norway; 5 Department of Research and Innovation, Division of Clinical Neuroscience, Oslo University Hospital, Oslo, Norway; 6 Institute of Environmental Medicine, Intervention and implementation research for worker health, Karolinska Institutet, Stockholm, Sweden

**Keywords:** Back pain, Symptom satisfaction, Clinical course, Older adults

## Abstract

**Background:**

Back pain is known to increase with age. However, most studies are conducted in the younger adult population, leaving the older adults understudied. The clinical course and associated prognostic factors for back pain remain poorly investigated in this population. Hence, this study aimed to investigate the clinical course of back pain and symptom satisfaction across groups derived from a latent class analysis in older adults: the Positive, the Fearful, the Distressed, and the Hopeful, over a 2-year period.

**Methods:**

Participants were ≥ 55 years of age, seeking primary care due to an episode of back pain. Observational data from baseline and 2-year follow-up was used. Due to similar characteristics between the Fearful and the Distressed groups they were combined. Linear mixed model analyses were conducted, comparing the effect of time and group on pain intensity and symptom satisfaction.

**Results:**

A total of 417 and 275 participants were included at baseline and the 2-year follow-up, respectively. The Positive had a generally more favourable clinical course, the Hopeful showing slightly worse, and the Fearful & Distressed showing the worst scores. However, all groups showed similar levels of pain intensity and symptom satisfaction at the 2-year end point.

**Conclusion:**

Patients with a positive attitude showed lower levels of pain intensity and improved levels of symptom satisfaction throughout most of the study compared to those who were fearful and distressed. Further investigations are needed to explore the effect of actively pursuing a positive outlook during the course of back pain in older adults.

**Supplementary Information:**

The online version contains supplementary material available at 10.1186/s12891-025-09442-w.

## Introduction

Back pain is an increasing global burden [1–4], and has been deemed the leading cause of disability in most countries [5]. As many as 80% of people will experience back pain at some point during their life [6], with a higher prevalence in women [5] and in older age groups, i.e., between 50 and 75 years of age [5, 7, 8]. Back pain may be due to or influenced by several factors, such as gender, body mass index, duration of pain, disability, and negative expectations of recovery, all found to predict an intermediate or high intensity back pain trajectory [9–13]. The literature shows an increasing number of studies being conducted on the trajectory of back pain in adult patients [9, 10, 12, 14, 15]. Despite knowing that older age is an important factor for the prevalence of back pain [7], such studies on older adults are few [10, 12]. However, more information on the different trajectories of back pain in older adults has been presented [3, 13, 16–18], which has uncovered between 3 and 6 subgroups of pain trajectories [9, 12, 16].

Moreover, the development of back pain during treatment, i.e., the clinical course, can vary considerably between individuals [19], but is often seen as a long-term and fluctuating condition [14], with periods of flare-ups and improvement [12]. This variation could be explained by biopsychosocial differences between individuals as well as the influence of time on the natural history of back pain. Similarly to the number of groups found in previous research on trajectories, work by our research group has identified four groups in older adults with back pain [20]. The four groups were labelled, “The Positive” (39%), “The Fearful” (7%), “The Distressed” (8%), “The Hopeful” (46%), respectively (see Supplementary Table 1 for summary of characteristics for each group). This latent class analysis was developed in a cross-sectional material of older adults seeking primary care due to back pain [20]. It is therefore of interest to investigate any potential difference in clinical course between these four groups in a prospective study.

A commonly used outcome measure of back pain, important for both clinicians and patients, is pain intensity. This can be measured with different tools representing different aspects of pain such as the numeric rating scale (NRS) [21]. Another outcome measure that can reflect a different aspect of pain is symptom satisfaction. In previous research participants have been asked about symptom satisfaction through a single item, patient acceptable symptom state (PASS) [22, 23]. However, to our knowledge there are no previous studies exploring the clinical course of both NRS and PASS in older adults with back pain. Our hypothesis is that there may be a difference in the clinical course of these two outcomes depending on previously determined group characteristics.

Hence, the aim of this study was to identify the 2-year clinical course of back pain intensity and symptom satisfaction in groups identified among older adults ≥ 55 years of age seeking primary care, i.e., general practitioners (GP), physiotherapists, and chiropractors, due to an episode of back pain.

## Methods

The PROGnosis RESearch Strategy (PROGRESS) type II framework was used when designing and executing this study [24]. In line with recommendations from the PROGRESS framework [24] a study protocol, including a statistical analysis plan was published February 23rd 2024 (registered at Clinicaltrials.gov, identifier NCT04261309) [25].

### Design and setting

This study presents data from the BAck Complaints in the Elderly—Norway study (BACE-N), a prospective observational cohort study with 2-years of follow-up within a Norwegian primary care setting BACE-N is a part of the international BACE consortium [26].

### Recruitment procedure and participants

The recruitment took place in primary care between April 2015 and February 2020. People ≥ 55 years of age seeking care at 110 general practitioners (GPs)s, physiotherapists, or chiropractors in Norway with a new episode of back pain were considered eligible for participation. Patients were excluded if they had sought health care for similar back pain within the previous 6 months, had difficulty completing the questionnaire due to language barriers, or had difficulty completing the physical examinations, included but not limited to, e.g., being wheelchair bound.

### Data collection, outcomes, and other variables

At baseline, the participants answered an extensive questionnaire and completed a standardized physical examination. At 3-, 6-, 12-, and 24-month follow-up questionnaires were distributed either electronically or by mail.

The outcomes of interest were back pain intensity over the course of the previous week measured by the Numeric Rating Scale (NRS) (range 0–10, higher score indicates higher back pain intensity) [27, 28], and symptom satisfaction measured by the PASS (range 1–5, higher score indicates increasing dissatisfaction) [22], consisting of a single item “How satisfied would you be if your current symptoms were to persist the rest of your life?” and categorised into: “very satisfied”, “somewhat satisfied”, “neither satisfied nor dissatisfied”, “somewhat dissatisfied” and “very dissatisfied”. NRS and PASS were measured at all time points.

Due to the confounding nature of the following variables both analyses were adjusted for age [12, 13, 29], gender [12, 30], educational level [31], employment status [32, 33] and visited primary health care practitioner [34].

### Latent classes

The present paper is based on four previously determined latent classes by our research group uncovered by use of a single-stage latent class analysis. The choice of model was based on several fit indices, such as Bayesian information criteria (BIC), Akaike information criteria (AIC), and posterior probability [20]. These groups were based on a range of pain and psychological variables measured at baseline (for detailed overview of these items please see Supplementary Table 1). The different pain measurements consisted of pain intensity (NRS), pain duration [27, 35], pain location (categorized McGill pain drawing [36]), and psychological aspects of experiencing pain such as pain catastrophising (using the Pain Catastrophising scale (PCS)) [37]. To measure disability and comorbidities, the Roland Morris Disability Questionnaire (RMDQ) [38] and the Self-Administered Comorbidity Questionnaire (SCQ) [39] were used, respectively. Measurements to assess kinesiophobia, beliefs, expectations and depression were also included using the Fear Avoidance Belief Questionnaire (FABQ-pa) [40], the Back Belief Questionnaire (BBQ) [41], expectations of back pain after 3 months and the Centre for Epidemiologic Studies Depression Scale (CES-D) [42], respectively. Lastly, inquiries on intake of medication were included. The resulting groups were named the Positive, the Fearful, The Hopeful and the Distressed. Based on the variables chosen in this study, most individuals (85%) were found to present favourable psychological and behavioural characteristics, especially the Positive and the Hopeful. However, belonging to the groups Fearful or Distressed showed overall worse scores on the chosen characteristics [20].

For the purpose of the present paper, the two groups the Fearful and the Distressed (*n* = 31 and *n* = 33, respectively), were combined to one group (the Fearful & Distressed) due to small numbers and characteristic similarities.

### Statistical analysis

To investigate the clinical course over 2 years of follow-up across the groups, a linear mixed model analysis was chosen due to its flexibility, allowance of more complex grouping structures, handling of missing data and both fixed and random effects [43]. Hence, no imputation was conducted for missing values. An unadjusted and a full model were tested for both outcomes. The full models were adjusted for baseline age, gender, education, employment, and the chosen first-contact provider. Margin plot based on the full models were created. The models included primary fixed effects (time, latent class and interaction between time and latent class), and a subject-specific random intercept. Post hoc analyses (pairwise comparisons of margins) were conducted to explore any statistically significant differences between all groups at the different time points for both outcomes. The analyses were performed using STATA/SE 17.0 ໿(StataCorp, College Station, TX, USA). A P-value < 0.05 was deemed statistically significant.

### Sample size

The sample size calculations are provided in the BACE-N protocol [25]. We considered a sample size of 450 as sufficient for the analyses conducted in the present paper [44].

## Results

**Fig. 1:**
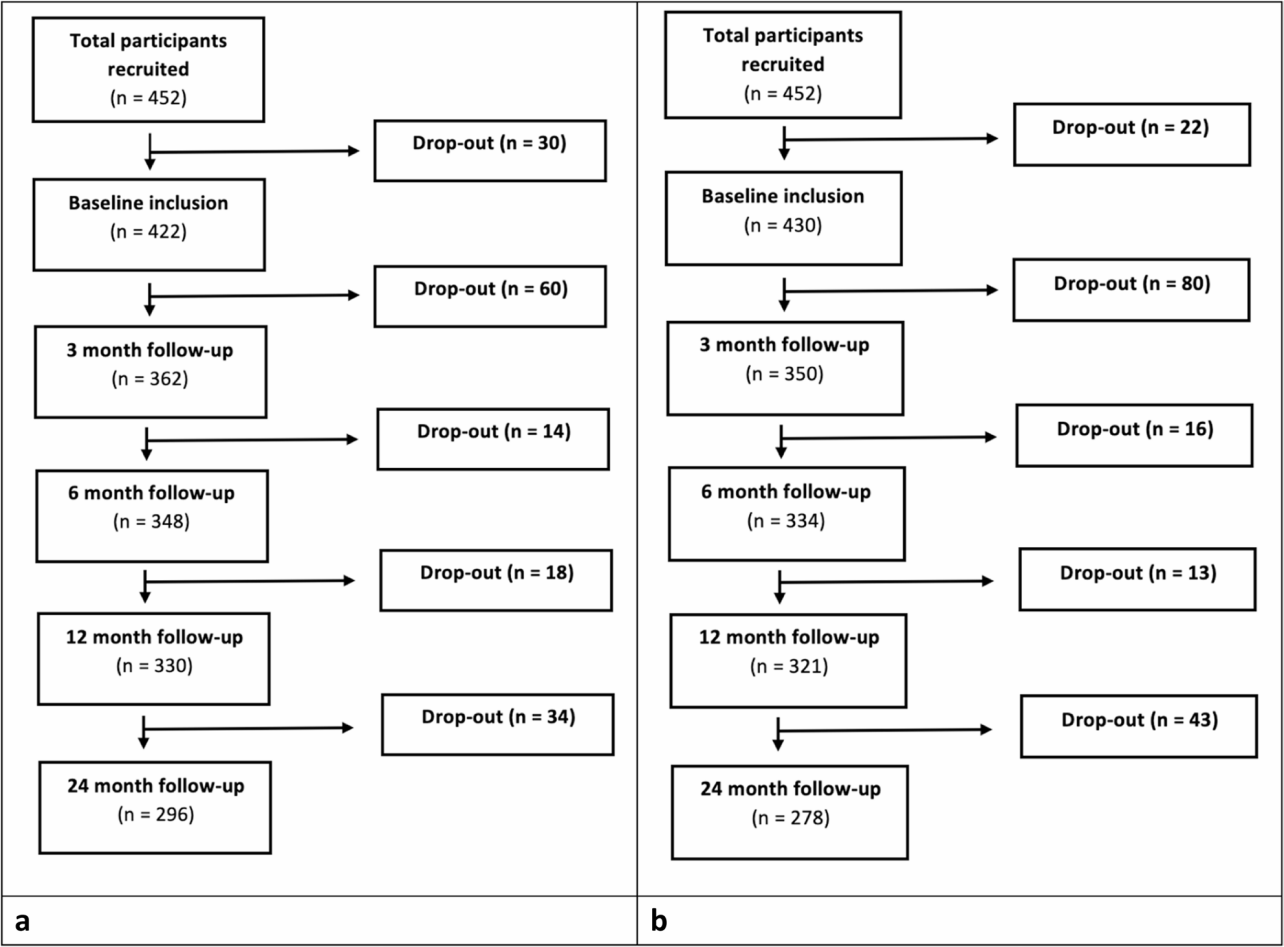
**A** Flowchart of the study for NRS that had also been assigned to LCA groups. **B** Flowchart of the study for PASS that had also been assigned to LCA groups.

A total of 452 participants were recruited (127 patients from GPs, 130 patients from physiotherapists, and 195 patients from chiropractors) of which a total of 422 (NRS) and 430 (PASS) participants were included in the analyses. These numbers were based on those participants who reported on the outcomes of interest at the different time points and were also assigned previously defined LCA groups [20]. There was some loss to follow-up during the 2-years, with a 29.8% for the NRS and 35.3% for the PASS (see Fig. 1a-b) from baseline to the end of the study. Reasons for drop out between baseline and 24 months follow-up were that some participants found the study to be not appropriate for them, difficulty answering questionnaires, pain free, other disease, unknown and death.

### Sample characteristics

Patient characteristics at baseline and 2-year follow-up are described in Table 1. The mean age of the cohort was 67 years, 52% were women, and the average pain intensity and symptom satisfaction at baseline was 6/10 and 4/5, respectively. Of the three groups, the Positive group showed lower pain levels, higher symptom satisfaction, and had the highest percentage of higher education at baseline. However, at the 24-month follow-up the differences in pain intensity and symptom satisfaction were reduced between the groups. Patient characteristics at baseline and 24-month follow-up, analyses and visualisation of results based on the four original groups can be seen in supplementary Tables 2, 3 and supplementary Fig. 1a & 1b.

**Table 1:** Patient characteristics at baseline and at 24 months stratified by LCA classes

	Baseline (n* = 417)			24-month follow-up (n* = 275)		
	Class 1 The Positive n* = 161	Class 2 The Fearful & Distressed n* = 59	Class 3 The Hopeful n* = 197	Class 1 The Positive n* = 105	Class 2 The Fearful & Distressed n* = 45	Class 3 The Hopeful n* = 125
Age (mean (SD))	65.9 (7.7)	68.9 (9.4)	66.5 (8.2)	68.0 (7.8)	69.9 (9.2)	68.3 (8.1)
missing	*0*	*0*	*0*			
Female (*n* (%))	84 (49.7)	45 (70.3)	102 (50.4)	66 (51.9)	31 (70.4)	78 (56.1)
missing	*0*	*0*	*0*			
Education (*n* (%))						
Less than High School	11 (6.5)	10 (15.8)	24 (12.0)	8 (6.3)	8 (18.1)	17 (12.3)
High School	76 (44.9)	26 (41.2)	97 (48.5)	55 (43.3)	19 (43.1)	66 (47.8)
Higher education < 4 years	43 (25.4)	15 (23.8)	48 (24.0)	35 (27.5)	9 (20.4)	35 (25.6)
Higher education > 4 years	39 (23.0)	12 (19.0)	31 (15.5)	29 (22.8)	8 (18.1)	20 (14.4)
missing	*0*	*1*	*2*			
Paid work, yes (*n* (%))	73 (48.3)	27 (55.1)	103 (59.2)	47 (39.1)	9 (24.3)	46 (36.2)
missing	*18*	*15*	*28*			
First health care provider (n (%))						
General practitioner	32 (19.1)	32 (19.1)	63 (31.2)	24 (19.2)	20 (45.4)	40 (27.2)
Physiotherapist	45 (26.9)	45 (26.9)	61 (30.2)	35 (28.0)	11 (25.0)	43 (29.0)
Chiropractor	90 (53.8)	90 (53.8)	78 (38.6)	66 (52.8)	13 (29.5)	56 (43.8)
missing	*2*	*0*	0			
Pain intensity (NRS) (mean (SD))	4.1 (2.2)	6.5 (1.9)	6.0 (2.0)	2.3 (2.7)	3.3 (3.1)	2.7 (2.6)
missing	12	6	11	54	*14*	*71*
Symptom satisfaction (PASS) (mean (SD))	3.3 (1.2)	4.5 (0.8)	4.2 (1.0)	2.6 (1.4)	2.8 (1.5)	2.6 (1.3)
missing	*2*	2	1	62	19	76

*SD* Standard Deviation, *n* total number derived from all participants, n* total number who reported on both outcomes, *NRS* Numeric Rating Scale, *PASS* Patient Acceptable Symptom State

**Fig. 2:**
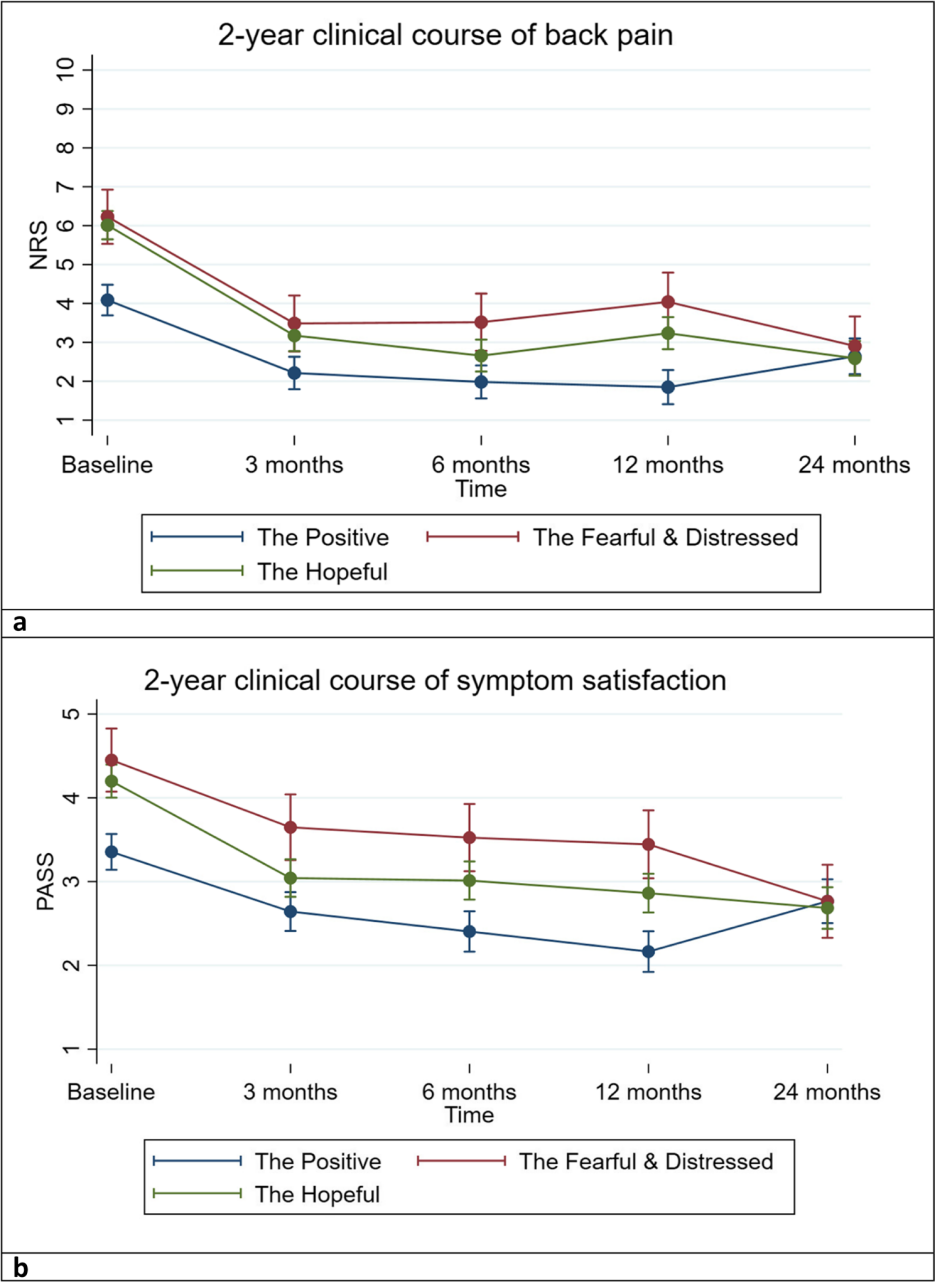
**A** Margins plot: Clinical course of back pain intensity from baseline to 24 months follow-up. NRS – Numeric Rating Scale. **B** Margins plot: Clinical course of symptom satisfaction from baseline to 24 months follow-up. PASS – Patient Acceptable Symptom State: 1) ”very satisfied”, 2) “somewhat satisfied”, 3) “neither satisfied nor dissatisfied”, 4) “somewhat dissatisfied” and 5) “very dissatisfied”.

The biggest improvement for both outcomes in all groups was seen between baseline and 3 months. From baseline a trend of decreasing pain intensity was seen primarily at 3 months with a relative stable course from 3 to 24 months for all groups. However, a slight increase in NRS scores was seen at 12 months for the Fearful & Distressed and the Hopeful, and at 24 months for the Positive (Fig. 2a). Also, an improvement of PASS scores were seen for all groups, up until 24 months where the Positive showed a slight decrease in PASS scores (Fig. 2b). The Positive group showed the most favourable clinical course for both outcomes during the first year of follow-up, but at 24 months both NRS and PASS scores were similar across the groups. Furthermore, the clinical course of pain intensity and symptom satisfaction were similar across the two groups Fearful & Distressed and Hopeful, with some minor variations (see interactions time x LCA in Table 2). Table 2 shows that compared to the Positive, the Hopeful group had statistically significant interactions for NRS scores, showing a reduction in pain intensity at 3, 6, and 24 months, and for PASS scores, showing an improvement in symptom satisfaction at 3 and 24 months. For the Fearful & Distressed group a statistically significant interaction effect was seen at 24 months for PASS scores, showing an improvement in symptom satisfaction, compared to the Positive.

The Positive showed the best scores for most of the study, i.e., the lowest pain intensity and the highest symptom satisfaction. The Hopeful group tended to show somewhat less favourable scores, and the Fearful & Distressed showed the least favourable scores of all groups. Post hoc comparisons between groups showed that for NRS scores statistically significant differences were seen between the Positive and the other groups at all time points except at the 24-month follow-up. At 6 months there was also a statistically significant difference between the Hopeful and the Fearful & Distressed (supplementary Table 4). For PASS scores, statistically significant differences were seen between the Positive and the other groups at all time points except at the 24-month follow-up. At 3-, 6- and 12 months there was also a statistically significant difference between the Hopeful and the Fearful & Distressed (supplementary Table 5).

**Table 2:** Linear mixed models showing the association between LCA class, pain intensity and symptom satisfaction

	Pain intensity (NRS) score			Symptom satisfaction (PASS) score		
	B	95% CI	*P*	B	95% CI	*P*
Unadjusted model						
Follow-up time points						
Baseline (ref)						
3 months	**−2.01**	−2.492 to −1.546	**< 0.001**	**−0.73**	−0.95 to −0.47	**< 0.001**
6 months	**−2.14**	−2.623 to −1.673	**< 0.001**	**−0.96**	−1.22 to −0.69	**< 0.001**
12 months	**−2.34**	−2.827 to −1.854	**< 0.001**	**−1.21**	−1.48 to −0.94	**< 0.001**
24 months	**−1.64**	−2.154 to −1.144	**< 0.001**	**−0.67**	−0.95 to −0.39	**< 0.001**
LCA class						
The Positive (ref)						
The Fearful & Distressed	**2.47**	1.762 to 3.186	**< 0.001**	**1.19**	0.81 to 1.58	**< 0.001**
The Hopeful	**1.89**	1.392 to 2.396	**< 0.001**	**0.86**	0.59 to 1.13	**< 0.001**
Interaction Time x LCA						
Baseline x The Positive (ref)						
3 months x The Fearful & Distressed	−0.67	−1.566 to 0.218	0.139	−0.16	−0.65 to 0.32	0.516
3 months x The Hopeful	**−0.78**	−1.432−0.144	**0.016**	**−0.48**	−0.84 to −0.13	**0.007**
6 months x The Fearful & Distressed	−0.45	−1.372 to 0.454	0.324	0.00	−0.49 to 0.51	0.971
6 months x The Hopeful	**−1.05**	−1.703 to −0.404	**0.001**	−0.17	−0.53 to 0.18	0.329
12 months x The Fearful & Distressed	−0.10	−1.039 to 0.830	0.826	0.21	−0.29 to 0.72	0.403
12 months x The Hopeful	−0.34	−1.010 to 0.310	0.299	−0.10	−0.46 to 0.25	0.570
24 months x The Fearful & Distressed	+**−1.59**	−2.527 to −0.653	**0.001**	**−1.06**	−1.58 to −0.53	**< 0.001**
24 months x The Hopeful	**−1.61**	−2.307 to −0.929	**< 0.001**	**−0.81**	−1.19 to −0.43	**< 0.001**
Full model						
Follow-up time points						
Baseline (ref)						
3 months	**−1.87**	−2.38 to −1.36	**< 0.001**	**−0.71**	−0.99 to −0.43	**< 0.001**
6 months	**−2.10**	−2.61 to−1.59	**< 0.001**	**−0.95**	−1.23 to −0.66	**< 0.001**
12 months	**−2.23**	−2.76 to −1.71	**< 0.001**	**−1.19**	−1.47 to −0.90	**< 0.001**
24 months	**−1.44**	−1.99 to −0.90	**< 0.001**	**−0.58**	−0.89 to −0.28	**< 0.001**
LCA class						
The Positive (ref)						
The Fearful & Distressed	**2.14**	1.33 to 2.94	**< 0.001**	**1.09**	0.66 to 1.53	**< 0.001**
The Hopeful	**1.92**	1.38 to 2.46	**< 0.001**	**0.84**	0.55 to 1.13	**< 0.001**
Interaction Time x LCA						
Baseline x The Positive (ref)						
3 months x The Fearful & Distressed	−0.86	−1.88 to 0.14	0.095	−0.09	−0.64 to 0.46	0.750
3 months x The Hopeful	**−0.96**	−1.66 to −0.26	**0.007**	**−0.44**	−0.83 to −0.05	**0.024**
6 months x The Fearful & Distressed	−0.60	−1.63 to 0.42	0.251	0.02	−0.53 to 0.58	0.931
6 months x The Hopeful	**−1.24**	−1.95 to −0.53	**0.001**	−0.23	−0.62 to 0.15	0.239
12 months x The Fearful & Distressed	0.05	−1.00 to 1.10	0.925	0.18	−0.38 to 0.75	0.524
12 months x The Hopeful	−0.53	−1.25 to 0.18	0.143	−0.14	−0.54 to 0.24	0.468
24 months x The Fearful & Distressed	**−1.87**	−2.94 to −0.80	**0.001**	**−1.09**	−1.69 to −0.49	**< 0.001**
24 months x The Hopeful	**−1.97**	−2.72 to −1.22	**< 0.001**	**−0.92**	−1.34 to −0.50	**< 0.001**

*B* regression coefficient, *Pp*-value, *CI* confidence interval, *NRS *Numeric Rating Scale, *PASS* Patient Acceptable Symptom State, bold numbers indicate statistical significance

Full model was adjusted for age, gender, education level, employment, and visit to chosen primary health care practitioner

## Discussion

This paper presents the clinical course of back pain intensity and symptom satisfaction over a 2-year period in three groups of participants aged 55 or older who sought primary care due to back pain in Norway. Despite all groups showing a general improvement some differences were seen. At baseline the Positive group initially presented better scores for both outcomes, whereas the two other groups, the Hopeful and the Fearful & Distressed, started at relatively similar scores for both pain intensity and symptom satisfaction. Throughout most of the study period the Positive group had a generally more favourable clinical course, with the Hopeful group showing slightly worse scores, and the Fearful & Distressed group showing the worst scores of the three groups. All groups showed the largest improvement for both outcomes at 3 months, which is in line with previous findings showing the biggest improvement in pain and disability between baseline and 3 months [16]. The Positive group, despite showing a trend of improvement for both outcomes was the only group that showed a slight increase in pain intensity and reduction in symptom satisfaction at the 2-year follow-up. Interestingly, all groups presented similar scores for both outcomes at the study end.

The experience of back pain is highly complex and is influenced by different factors ranging from purely biological and structural to a variety of psychosocial factors [2]. Our results showed that patient characteristics, consisting of a variety of attitudes and beliefs, seemed to be influential factors for the clinical course of both back pain and symptom satisfaction in older adults. As mentioned, the Positive group showed a more favourable clinical course compared to the two other groups up until the 2-year end point. This group generally had the lowest pain intensity, disability, depression and catastrophising score, in addition to the lowest number of comorbidities [20]. These findings seem to be in line with previous research showing that more positive beliefs, attitudes and expectations have an impact on the development and coping with pain [45–47], which could also lead to a reduction in pain intensity [45]. Moreover, having a more cheerful view on the situation [48, 49] and being less troubled by e.g., depression [50, 51], is known to have a positive impact on pain levels. The Hopeful group showed the second most favourable clinical course. They had slightly higher scores for disability, fear avoidance behaviour, depression and catastrophising compared to the Positive group, which might be an explanation as to why they had slightly higher pain intensity levels throughout the follow-up period. Interestingly, the Hopeful group and the Fearful & Distressed group had a similar pain intensity score at baseline, but the Hopeful group had consistently lower pain intensity scores throughout the study compared to the Fearful & Distressed group. This might further support the evidence that more optimistic attitudes and beliefs can have a positive impact on pain intensity. In contrast to the Hopeful group, the Fearful & Distressed group, combined, had the highest scores for pain intensity, fear avoidance behaviour, disability, depression, catastrophising and comorbidities. These results are in line with previously identified associations indicating that unfavourable characteristics such as increased pain catastrophising [52–54] and negative beliefs [55] have been linked to negative outcomes. However, there might be a bidirectional relationship of certain personality traits and pain intensity [56], e.g., those with higher levels of low back pain have been found to be more likely to hold more negative beliefs [55]. Moreover, due to the relatively long follow-up period of this study it is not unlikely that some participants might have had a change in attitudes and beliefs over time. Such change could potentially, at least partially, explain why all groups presented with similar pain intensity levels at the 2-year end point. Though this could also be influenced by other factors such as the fluctuating nature of back pain, and/or other psychosocial factors not included in the present study.

As with pain intensity, the Positive group was the only group that presented with a slight worsening of symptom satisfaction at the 2-year follow-up. The similar trend in clinical course for the three groups regarding both outcomes could indicate a relationship between symptom satisfaction and pain intensity, or that similar factors might influence both outcomes. Factors like age, low level of pain intensity and better emotional well-being has been indicated as important factors for increased, i.e., improved, symptom satisfaction [57]. In the present study, the group with the biggest improvement in symptom satisfaction was the Fearful & Distressed group. Despite starting off with the least advantageous characteristics, one could speculate that this group would have the biggest room for improvement. Characteristics such as high fear-avoidance, comorbidity, and symptoms of depression, may have been more present at the beginning of a back pain episode and may have gradually lessened over time, which in turn could explain this improvement in symptom satisfaction. The change may also have been influenced by number of treatments and other psychosocial factors changing or improving over this 2-year period.

Importantly, previous studies have found that, among older adults, back pain has been associated with perceived difficulty in performing tasks, activities of daily living, and physical capacities [3, 58, 59]. Moreover, an increased risk of frailty [60] and reduced balance in older adults with back pain [61] has also been indicated. In an age group where such activities may be related to quality of life, social interactions, and wellness it seems important to increase our understanding of pain and its consequences in this age group. Furthermore, persistent pain is increasingly more prevalent in the older adult population [62, 63], hence achieving an acceptable level of symptom satisfaction is likely especially important for this population as any improvement may increase quality of life. If a pain-free state is unlikely to achieve for certain individuals, one could argue that a shift in focus towards strategies of coping and achieving quality of life with, or despite of, the pain would be more beneficial. Thus, more research is needed to understand the true significance of patient characteristics, such as attitudes and beliefs, and their impact and influence on the course of back pain and symptom satisfaction in the older adult population.

### Strengths and limitations

One of the strengths of this study was the long follow-up period with frequent assessment of the outcomes. This provided an opportunity to explore the long-term course of pain intensity and symptom satisfaction in a less investigated population. However, the chosen follow-up points could also be considered a limitation. Due to the relatively long periods between some of the follow-ups (e.g., between 1 and 2 years), any information on pain experiences between these time points is not known. It may be that some participants experienced persistent pain and others recurrent pain, details which were not captured by our measurements. More frequent measurements would have provided more accurate information about the variation in clinical course. Further, as with most long-term follow-up studies, drop out and a reduction in response rates is to be expected. For most of the non-responders the reasons for lack of response are not known. However, reasons for drop out was reported to be e.g., other diseases, difficulty answering questionnaires, being pain free, and death. Even though previous work by our group showed that gender was the only characteristic that differentiated the responders from the non-responders at 6- and 12 months, it might be that those participants that dropped out or did not respond at 24 months were different from those who remained in the study, hence potential attrition bias cannot be excluded.

Moreover, the groups used in the present study was based on previous work by our research group. One important limitation is that in the construction of the groups NRS at baseline was included. Even though this is not the same measurement as our outcome measurements at the later time points it might have influenced the current findings. Moreover, in combining the groups Fearful and Distressed some nuance might have been lost. However, these two groups showed mostly similar characteristics. Of the 11 variables 8 (NRS, widespread pain, RMDQ, FABQ, expectation to recovery, medication use and duration of pain) were the same or similar between the groups. The 3 variables that showed some difference in distribution were other comorbidities (Fearful presented more), BBQ score (Fearful presented more) and CES-D (Distressed presented more). Additionally, after assessing the highly similar clinical course of the four groups (shown in supplementary Fig. 1a and 1b), a decision was made that presenting three groups would be the more appropriate. Despite a potential loss of nuance, the robustness of the statistical analysis would increase, providing slightly more reliable results.

The observed reduction in pain intensity and improvement in symptom satisfaction may be due to the natural history of back pain or it may be due to the received treatment after inclusion into the cohort study. Even though the model was adjusted for visit to certain health care professionals, the influence and importance of treatment type and frequency needs to be investigated in future studies. Further, in this study the structural origin of back pain was not known, which may also have impacted the clinical course. Hence, further studies which also includes the specific origin of back pain and to what extent that influences the long-term outcome is warranted.

Further, the statistical analyses chosen for the present paper may also have limitations. Different results may have presented if other methods were used, such as latent profile analyses or other forms of repeated measure analyses. However, using a linear mixed model has the advantage of being more robust in terms of missing data, which is important to consider in any longitudinal study. Moreover, it also has the advantage of assessing both within and between difference which was of interest in the present study.

### Clinical implications

The implications of characterising back pain patients with higher precision may have importance for treatment focus and strategies e.g., to prevent further disability and reduction in quality of life. Creating subgroups and investigating their clinical course has the potential to be of clinical value. For the patient group assessed in the present paper an overview of how their back pain intensity and symptom satisfaction presents over time has the potential to inform about long-term prognosis. However, prior to concluding whether our results have any such value more research is needed. Future research should investigate whether the groups have a predictive value, and if so to what extent. This can be done by prognostic models, which in turn would require internal and external validation to ensure transferability and generalizability. Hence, based on the results in the present paper, specific clinical implications are uncertain but there is potential to utilize the groups for further research.

## Conclusion

The present study revealed the clinical course of pain intensity and symptom satisfaction in three groups of older adults with back pain seeking primary care. Patients with a positive attitude showed lower levels of pain intensity and improvement of symptom satisfaction during the first year of follow-up as compared to those who were fearful and distressed. However, all three groups showed similar scores for both outcomes at the 2-year endpoint. Further investigations are needed to explore the true influences of these characteristics and the effect of actively pursuing a positive outlook during the course of back pain in the older adults.

## Supplementary Information

Supplementary Material 1. 

## Data Availability

All data relevant to the study are included in the article or uploaded as online supplemental information.
