# Supplementary material for: Two-years clinical course of pain intensity and symptom satisfaction for latent classes in older adults with back pain
Source: BMC Musculoskelet Disord. 2025 Dec 23;27:62. doi: 10.1186/s12891-025-09442-w (PMC12837090; doi:10.1186/s12891-025-09442-w)
Supplement: Supplementary file 1 — Supplementary Material 1. [file 12891_2025_9442_MOESM1_ESM.docx]

| **Supplementary table 1. Summary of group characteristics and detailed information on questionnaire items used to develop the previously determined four latent classes (22).** | | | | | | |
| --- | --- | --- | --- | --- | --- | --- |
| **Latent class label** | **The Positive (39%)** | | **The Fearful (7%)** | | **The Distressed (8%)** | **The Hopeful (46%)** |
| **Summary of characteristics** | The overall lowest score for reported pain intensity, disability, comorbidities, symptoms of depression, and pain catastrophising. | | The highest score on fear avoidance behaviour and comorbidity, with the second highest score on reported pain intensity, disability, catastrophising, and symptoms of depression. | | The highest score on pain intensity, disability, symptoms of depression, and pain catastrophising. They also had the second highest score on both reported comorbidities and fear avoidance behaviour. | The second lowest overall score on all measurements. Unlike The Positive they had somewhat higher scores on pain intensity, disability, fear avoidance behaviour, symptoms of depression, and pain catastrophising. |
| **Questionnaire** | | **Range** | | **Score interpretation** | | |
| Back pain intensity (NRS) | | 0-10 | | Higher number indicate higher pain intensity | | |
| Duration of back pain | | 0-14 days, 15-90 days, 91-365 days or ≥366 days | | - | | |
| Widespread pain | | 0-15 | | Higher score indicates higher number of pain sites | | |
| Medication use | | Yes or no | | - | | |
| Number of comorbidities (SCQ) | | 0-7 | | Higher number indicate higher number of comorbidities | | |
| Disability (RMDQ) | | 0-24 | | Higher score indicates more back-related disability | | |
| Kinesiophobia (FABQ-PA) | | 0-24 | | Higher score indicates higher level of kinesiophobia | | |
| Catastrophising (PCS) | | 0-52 | | Higher score indicates more pain catastrophising | | |
| Back pain beliefs (BBQ) | | 9-45 | | Higher score indicates more pessimistic beliefs regarding the consequences of back pain | | |
| Symptoms of depression (CES-D) | | 0-60 | | Higher scores indicate the presence of more symptomatology | | |
| Expectation | | Better/much better or no change/worse | | - | | |
| Characteristics were based on the following measurements:  **NRS** - Numeric rating scale for pain intensity, **Widespread pain** - pain location (categorized McGill pain drawing), **SCQ** - Self-Administered Comorbidity Questionnaire, **RMDQ** - Roland Morris Disability Questionnaire, **FABQ -PA** - Fear Avoidance Belief Questionnaire-Physical activity subscale, **PCS** - Pain Catastrophising scale, **BBQ** - Back Belief Questionnaire, **CES-D** - Centre for Epidemiologic Studies Depression Scale and **Expectation** - expectations of back after 3 months. | | | | | | |

| **Supplementary table 2. Patient Characteristics at baseline and at 24 months stratified by LCA classes** | | | | | | | | |
| --- | --- | --- | --- | --- | --- | --- | --- | --- |
|  | **Baseline (n* = 435)** | | | | **24-month follow-up (n* = 234)** | | | |
|  | **Class 1**  **“The Positive”**  **n* = 169** | **Class 2**  **“The Fearful”**  **n* = 31** | **Class 3**  **“The Distressed”**  **n* = 33** | **Class 4**  **“The Hopeful”**  **n* = 202** | **Class 1**  **“The**  **Positive”**  **n* = 92** | **Class 2**  **“The Fearful”**  **n* = 16** | **Class 3**  **“The Distressed”**  **n* = 18** | **Class 4**  **“The Hopeful”**  **n* = 102** |
| **Age (mean (SD))** | 65.9 (7.7) | 72.2 (9.5) | 65.7 (8.3) | 66.5 (8.2) | 68.1 (7.8) | 73.6 (9.6) | 66.7 (7.5) | 68.3 (8.1) |
| **Female (n (%))** | 84 (49.7) | 22 (70.9) | 23 (69.9) | 102 (50.4) | 66 (51.9) | 15 (71.4) | 16 (69.5) | 78 (56.1) |
| **Education (n (%))**  Less than High School  High School  Higher education <4 years  Higher education >4 years | 11 (6.5)  76 (44.9)  43 (25.4)  39 (23.0) | 2 (9.6)  11 (35.4)  11 (35.4)  6 (19.3) | 7 (21.8)  15 (46.8)  4 (12.5)  6 (18.7) | 24 (12.0)  97 (48.5)  48 (24.0)  31 (15.5) | 8 (6.3)  55 (43.3)  35 (27.5)  29 (22.8) | 3 (14.2)  8 (38.1)  6 (28.5)  4 (19.0) | 5 (21.7)  11 (47.8)  3 (13.0)  4 (17.3) | 17 (12.3)  66 (47.8)  35 (25.6)  20 (14.4) |
| **Paid work, yes (n (%))** | 73 (48.3) | 13 (54.1) | 14 (56.0) | 103 (59.2) | 47 (39.1) | 4 (23.5) | 5 (25.0) | 46 (36.2) |
| **First health care provider (n (%))**  General practitioner  Physiotherapist Chiropractor | 32 (19.1)  45 (26.9)  90 (53.8) | 14 (45.1)  9 (29.0)  8 (25.8) | 14 (42.4)  11 (33.3)  8 (24.2) | 63 (31.2)  61 (30.2)  78 (38.6) | 24 (19.2)  35 (28.0)  66 (52.8) | 8 (38.1)  6 (28.6)  7 (33.3) | 12 (52.2)  5 (21.7)  6 (26.1) | 40 (27.2)  43 (29.0)  56 (43.8) |
| **Pain intensity (NRS)  (mean (SD))** | 4.1 (2.2) | 6.5 (1.9) | 6.5 (1.9) | 6.0 (2.0) | 2.39 (2.7) | 2.9 (2.7) | 3.8 (2.9) | 2.7 (2.6) |
| **Symptom satisfaction (PASS) (mean (SD))** | 3.3 (1.2) | 4.4 (1.0) | 4.6 (0.7) | 4.2 (1.0) | 2.6 (1.4) | 2.7 (1.4) | 2.9 (1.5) | 2.6 (1.3) |
| **SD** – standard deviation, **n** – number, **n*** - total number without any missing variables, **NRS** – Numeric Rating Scale, **PASS** – Patient Acceptable Symptom State | | | | | | | | |

| **Supplementary table 3. Estimates of fixed effects parameters from linear mixed models. Showing the association between LCA class, pain intensity (NRS) and symptom satisfaction (PASS) over 24 months.** | | | | | | |
| --- | --- | --- | --- | --- | --- | --- |
|  | **NRS score** | | | **PASS score** | | |
|  | **B** | **95% CI** | ***P*** | **B** | **95% CI** | ***P*** |
| **Unadjusted model** |  | |  |  | | |
| **Follow-up time points**  Baseline (ref)  3 months  6 months  12 months  24 months | **-2.019**  **-2.148**  **-2.341**  **-1.649** | -2.491 to -1.547  -2.622 to -1.673  -2.827 to -1.855  -2.154 to -1.144 | **<0.001**  **<0.001**  **<0.001**  **0.001** | **-0.734**  **-0.963**  **-1.214**  **-0.672** | -0.955 to -0.475  -1.228 to -0.698  -1.481 to -0.946  -0.955 to -0.390 | **<0.001**  **<0.001**  **<0.001**  **<0.001** |
| **LCA class**  The Positive (ref)  The Fearful  The Distressed  The Hopeful | **2.420**  **2.522**  **1.894** | 1.478 to 3.363  1.594 to 3.449  1.392 to 2.396 | **<0.001**  **<0.001**  **<0.001** | **1.066**  **1.321**  **0.862** | 0.557 to 1.575  0.825 to 1.817  0.593 to 1.132 | **<0.001**  **<0.001**  **<0.001** |
| **Interaction Time x LCA**  Baseline x The Positive (ref)  3 months x The Fearful  3 months x The Distressed  3 months x The Hopeful  6 months x The Fearful  6 months x The Distressed  6 months x The Hopeful  12 months x The Fearful  12 months x The Distressed  12 months x The Hopeful  24 months x The Fearful  24 months x The Distressed  24 months x The Hopeful | -0.888  -0.445  **-0.788**  -0.374  -0.516  **-1.054**  -0.503  0.247  -0.349  **-1.894**  **-1.255**  **-1.618** | -2.052 to 0.275  -1.620 to 0.729  -1.431 to -0.145  -1.601 to 0.852  -1.692 to 0.659  -1.703 to -0.405  -1.764 to 0.757  -0.953 to 1.448  -1.009 to 0.310  -3.107 to -0.682  -2.491 to -0.019  -2.306 to -0.930 | 0.135  0.457  **0.016**  0.550  0.389  **0.001**  0.434  0.686  0.299  **0.002**  **0.047**  **<0.001** | -0.174  -0.139  **-0.486**  0.157  -0.124  -0.179  0.414  0.041  -0.105  **-1.038**  **1.083**  **-0.816** | -0.814 to 0.466  -0.778 to 0.499  -0.841 to -0.130  -0.517 to 0.832  -0.766 to 0.518  -0.539 to 0.180  -0.239 to 1.097  -0.612 to 0.695  -0.469 to 0.258  -1.735 to -0.340  -1.764 to -0.402  -1.199 to -0.432 | 0.594  0.669  **0.007**  0.648  0.705  0.329  0.235  0.901  0.570  **0.004**  **0.002**  **<0.001** |
| **Full model** | | | | | | |
| **Follow-up time points**  Baseline (ref)  3 months  6 months  12 months  24 months | **-1.874**  **-2.105**  **-2.238**  **-1.446** | -2.382 to -1.367  -2.618 to -1.592  -2.763 to -1.713  -1.989 to -0.903 | **<0.001**  **<0.001**  **<0.001**  **<0.001** | **-0.712**  **-0.950**  **-1.190**  **-0.589** | -0.990 to -0.434  -1.236 to -0.664  -1.477 to -0.903  -0.893 to -0.284 | **<0.001**  **<0.001**  **<0.001**  **<0.001** |
| **LCA class**  The Positive (ref)  The Fearful  The Distressed  The Hopeful | **2.140**  **2.134**  **1.924** | 1.070 to 3.209  1.075 to 3.194  1.386 to 2.463 | **<0.001**  **<0.001**  **<0.001** | **0.977**  **1.206**  **0.844** | 0.395 to 1.560  0.637 to 1.774  0.552 to 1.135 | **0.001**  **<0.001**  **<0.001** |
| **Interaction Time x LCA**  Baseline x The Positive (ref)  3 months x The Fearful  3 months x The Distressed  3 months x The Hopeful  6 months x The Fearful  6 months x The Distressed  6 months x The Hopeful  12 months x The Fearful  12 months x The Distressed  12 months x The Hopeful  24 months x The Fearful  24 months x The Distressed  24 months x The Hopeful | -0.958  -0.772  **-0.962**  -0.553  -0.646  **-1.247**  -0.445  0.486  -0.537  **-2.334**  -1.373  **-1.975** | -2.295 to 0.377  -2.129 to 0.585  -1.662 to -0.261  -1.929 to 0.823  -2.005 to 0.713  -1.957 to -0.537  -1.865 to 0.974  -0.877 to 1.851  -1.256 to 0.182  -3.721 to -0.947  -2.800 to 0.053  -2.726 to -1.223 | 0.160  0.265  **0.007**  0.431  0.352  **0.001**  0.539  0.484  0.144  **0.001**  0.059  **<0.001** | 0.068  -0.246  **-0.445**  0.215  -0.158  -0.236  0.294  0.081  -0.146  **-1.336**  **-0.892**  **-0.925** | -0.662 to 0.800  -0.979 to 0.486  -0.830 to -0.059  -0.536 to 0.968  -0.894 to 0.577  -0.628 to 0.156  -0.468 to 1.057  -0.655 to 0.817  -0.541 to 0.249  -2.143 to -0.529  -1.666 to -0.119  -1.344 to -0.506 | 0.854  0.510  **0.024**  0.574  0.673  0.238  0.450  0.829  0.468  **0.001**  **0.024**  **<0.001** |
| **B** – regression coefficient, **P** – p-value, **CI** – confidence interval, **NRS** – Numeric Rating Scale, **PASS** – Patient Acceptable Symptom State, bold numbers indicate statistical significance. Full model was adjusted for age, gender, education level, employment, and visit to chosen primary health care practitioner. | | | | | | |

| 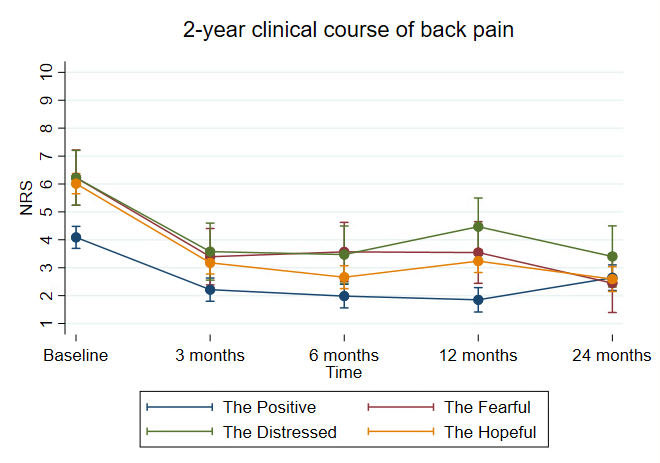 |
| --- |
| **Supplementary figure 1a.** Margins plot: Clinical course of back pain intensity from baseline to 24 months follow-up. NRS – Numeric Rating Scale. |
| **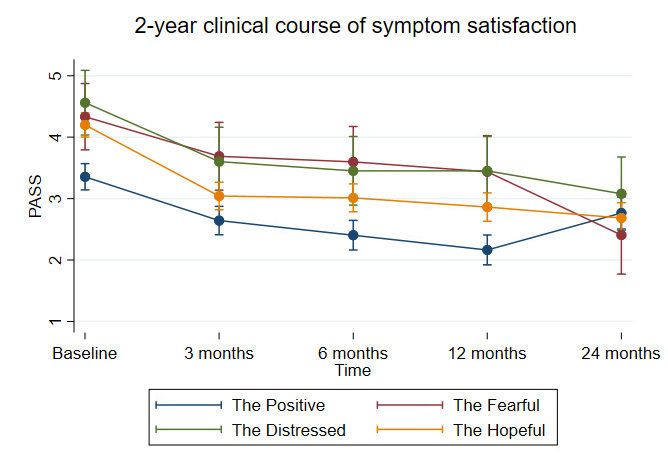** |
| **Supplementary figure 1b.** Margins plot: Clinical course of symptom satisfaction from baseline to 24 months follow-up. PASS – Patient Acceptable Symptom State: 1) ”very satisfied”, 2) “somewhat satisfied”, 3) “neither satisfied nor dissatisfied”, 4) “somewhat dissatisfied” and 5) “very dissatisfied”. |

| **Supplementary figure 4. Comparison between groups at all time points for NRS** | | | |
| --- | --- | --- | --- |
| **Group vs group** | **Contrast** | **Delta-method std err.** | **p-value** |
| **Baseline** |  | | |
| 2 vs 1 | 2.14 | 0.41 | 0.000 |
| 3 vs 1 | 1.92 | 0.27 | 0.000 |
| 3 vs 2 | -0.21 | 0.40 | 0.588 |
|  |  |  |  |
| **3 months** |  | | |
| 2 vs 1 | 1.27 | 0.42 | 0.003 |
| 3 vs 1 | 0.96 | 0.29 | 0.001 |
| 3 vs 2 | -0.31 | 0.41 | 0.459 |
|  |  |  |  |
| **6 months** |  | | |
| 2 vs 1 | 1.53 | 0.43 | 0.000 |
| 3 vs 1 | 0.67 | 0.30 | 0.025 |
| 3 vs 2 | -0.85 | 0.42 | 0.045 |
|  |  |  |  |
| **12 months** |  |  |  |
| 2 vs 1 | 2.19 | 0.44 | 0.000 |
| 3 vs 1 | 1.38 | 0.30 | 0.000 |
| 3 vs 2 | -0.80 | 0.43 | 0.065 |
|  |  |  |  |
| **24 months** |  |  |  |
| 2 vs 1 | 0.26 | 0.45 | 0.559 |
| 3 vs 1 | -0.04 | 0.32 | 0.879 |
| 3 vs 2 | -0.31 | 0.44 | 0.483 |
| 1 = The Positive, 2 = The Fearful & Distressed, 3 = The Hopeful | | | |

| **Supplementary figure 5. Comparison between groups at all time points for PASS** | | | |
| --- | --- | --- | --- |
| **Group vs group** | **Contrast** | **Delta-method std err.** | **p-value** |
| **Baseline** |  | | |
| 2 vs 1 | 1.09 | 0.22 | 0.000 |
| 3 vs 1 | 0.84 | 0.14 | 0.000 |
| 3 vs 2 | -0.25 | 0.21 | 0.247 |
|  |  |  |  |
| **3 months** |  | | |
| 2 vs 1 | 1.00 | 0.23 | 0.000 |
| 3 vs 1 | 0.39 | 0.16 | 0.015 |
| 3 vs 2 | -0.60 | 0.23 | 0.009 |
|  |  |  |  |
| **6 months** |  | | |
| 2 vs 1 | 1.12 | 0.23 | 0.000 |
| 3 vs 1 | 0.60 | 0.16 | 0.000 |
| 3 vs 2 | -0.51 | 0.23 | 0.029 |
|  |  |  |  |
| **12 months** |  |  |  |
| 2 vs 1 | 1.28 | 0.24 | 0.000 |
| 3 vs 1 | 0.69 | 0.17 | 0.000 |
| 3 vs 2 | -0.58 | 0.23 | 0.014 |
|  |  |  |  |
| **24 months** |  |  |  |
| 2 vs 1 | -0.00 | 0.26 | 0.999 |
| 3 vs 1 | -0.08 | 0.18 | 0.660 |
| 3 vs 2 | -0.08 | 0.25 | 0.753 |
| 1 = The Positive, 2 = The Fearful & Distressed, 3 = The Hopeful | | | |
